# Brequinar inhibits African swine fever virus replication in vitro by activating ferroptosis

**DOI:** 10.1186/s12985-023-02204-x

**Published:** 2023-10-24

**Authors:** Yang Chen, Yanchen Guo, Hao Chang, Zebu Song, Zhi Wei, Zhao Huang, Zezhong Zheng, Guihong Zhang, Yankuo Sun

**Affiliations:** 1 Guangdong Provincial Key Laboratory of Zoonosis Prevention and Control, College of Veterinary Medicine, South China Agricultural University, Guangzhou, China; 2 African Swine Fever Regional Laboratory of China (Guangzhou), Guangzhou, China; 3 Maoming Branch, Guangdong Laboratory for Lingnan Modern Agriculture, Maoming, Guangdong China; 4 Key Laboratory of Animal Vaccine Development, Ministry of Agriculture and Rural Affairs, Guangzhou, China

**Keywords:** African swine fever virus, Brequinar, Ferroptosis, Uridine

## Abstract

**Background:**

African swine fever virus (ASFV) is one of the most fatal swine etiological agents and has a huge economic impact on the global pork industry. Given that no effective vaccines or anti-ASFV drugs are available, there remains a pressing need for novel anti-ASFV drugs. This study aimed to investigate the anti-African swine fever virus (ASFV) activity of brequinar, a DHODH inhibitor.

**Methods:**

The anti-ASFV activity of brequinar was investigated using IFA, HAD, HAD_50_, qRT-PCR, and western blotting assays. The western blotting assay was used to investigate whether brequinar inhibits ASFV replication by killing ASFV particles directly or by acting on cell factors. The confocal microscopy and western blotting assays were used to investigate whether brequinar inhibits ASFV replication by activating ferroptosis.

**Results:**

In this study, brequinar was found to effectively inhibit ASFV replication ex vivo in porcine alveolar macrophages (PAMs) in a dose-dependent manner. In kinetic studies, brequinar was found to maintain ASFV inhibition from 24 to 72 hpi. Mechanistically, the time-of-addition assay showed that brequinar exerted anti-ASFV activity in all treatment modes, including pre-, co-, and post-treatment rather than directly killing ASFV particles. Notably, FerroOrange, Mito-FerroGreen, and Liperfluo staining experiments showed that brequinar increased the accumulation of intracellular iron, mitochondrial iron, and lipid peroxides, respectively. Furthermore, we also found that ferroptosis agonist cisplatin treatment inhibited ASFV replication in a dose-dependent manner and the inhibitory effect of brequinar on ASFV was partially reversed by the ferroptosis inhibitor ferrostatin-1, suggesting that brequinar activates ferroptosis to inhibit ASFV replication. Interestingly, exogenous uridine supplementation attenuated the anti-ASFV activity of brequinar, indicating that brequinar inhibits ASFV replication by inhibiting DHODH activity and the depletion of intracellular pyrimidine pools; however, the induction of ferroptosis by brequinar treatment was not reversed by exogenous uridine supplementation, suggesting that brequinar activation of ferroptosis is not related to the metabolic function of pyrimidines.

**Conclusions:**

Our data confirm that brequinar displays potent antiviral activity against ASFV in vitro and reveal the mechanism by which brequinar inhibits ASFV replication by activating ferroptosis, independent of inhibiting pyrimidine synthesis, providing novel targets for the development of anti-ASFV drugs.

**Supplementary Information:**

The online version contains supplementary material available at 10.1186/s12985-023-02204-x.

## Background

African swine fever (ASF) is a major economically important infectious disease caused by the African swine fever virus (ASFV), which threatens the global pork industry and has high mortality rate [1]. In the 1920s, ASF was first reported in Kenya and was limited to Africa [2]. In the 1950s, it spread to Europe, including Spain, Portugal, Italy, and France [3]. Europe (except for Sardinia) has eradicated ASFV using drastic control and eradication programs. Unfortunately, the disease reemerged in the Caucasus region in 2007 and rapidly spread to the eastern territory of the European Union in 2014 [4]. Next, the disease was reported on August 3, 2018, in China, one of the largest pork industries in the world. Between 2018 and 2022, 204 ASF outbreaks across 32 Chinese provinces were reported by the Chinese Ministry of Agriculture and Rural Affairs, causing huge economic losses, with estimates of at least 1.2 million sick and culled pigs [5, 6].

ASFV is the only member of the Asfarviridae family. It is a large, enveloped, double-stranded DNA virus, with 151–167 open reading frames, encoding more than 150 proteins [7]. ASFV is highly restricted to macrophages and monocytes, especially porcine alveolar macrophages (PAMs), which are the primary targets of ASFV in vivo [8]. Owing to the limited cell tropism and complex viral particle structure of ASFV, research on ASFV is exceedingly difficult, and there are no commercial vaccines or drugs to control ASFV infection [9]. So far, the main strategy to control ASF includes disinfection of vehicles and transit areas, strengthening of biosafety management on pig farms, and stricter vigilance programs [10]. Therefore, there is an exigent need to develop new methods to prevent ASFV infection, including vaccines and antiviral drugs.

In recent years, an increasing number of compounds with antiviral activity have been developed. For example, apigenin and genistein have been reported to exert inhibitory effects on the replication of ASFV [11, 12]. Mechanically, apigenin exerts inhibitory effects by impairing protein synthesis and viral factory formation. Furthermore, genistein acts as an ASFV-topo II poison, thereby inhibiting ASFV replication. GS-441524, an adenosine nucleoside analog, possesses significant anti-ASFV effects at a concentration of 200 μM by binding to the viral RNA, competing with the natural nucleoside ATP [13]. Other anti-ASFV inhibitors include resveratrol and oxyresveratrol [14], microalgae [15], cholesterol lowering drugs or inhibitors of cholesterol transport [16], antitumoral lauryl-gallate and anticonvulsivant valproic acid [17], calcium channels and SERMs [18], fluoroquinolones [19], even specific peptides [20, 21]. Significantly, drugs have the advantage of being multi-target antivirals, and targeting the host cell pathway may prevent the development of viral resistance to antiviral drugs.

Since host-supplied nucleoside biosynthesis is critical for viral replication, host enzymes involved in nucleoside biosynthesis and nucleotide biosynthesis pathways may serve as potential strategies for antiviral drug development. For example, Ribavirin, the most well-known antiviral drug, inhibits viral replication by inhibiting cellular IMP dehydrogenase, an enzyme required for guanine synthesis [22]. Dihydroorotate dehydrogenase (DHODH) is a fourth enzyme in the *de novo* pyrimidine biosynthesis pathway, thereby providing nucleotides for RNA/DNA synthesis essential for proliferation [23]. Brequinar as a DHODH inhibitor has been reported to inhibit viral replication by depleting intracellular pyrimidine pools, including Cantagalo virus [24], dengue virus [25], Ebola virus [26], foot-and-mouth disease virus [27], enterovirus [28] and the newly emerged coronavirus SARS-CoV-2 [29]. Strikingly, Mao and colleagues recently reported that brequinar activates ferroptosis by altering the ratio of ubiquinone to ubiquinol in a DHODH-dependent manner [30]. However, it is unknown whether brequinar inhibits viral replication through other mechanisms, in particular ferroptosis. In this study, we explored the antiviral activity of brequinar against ASFV infection. As expected, brequinar showed an outstanding inhibitory effect on ASFV infection by activating ferroptosis, which was not reversed by exogenous uridine supplementation.

## Materials and methods

### Cells and virus

PAMs were prepared from 4-week-old specific pathogen-free pigs, as previously described [31], and cultured in RPMI 1640 supplemented with L-glutamine (2 mM/mL), penicillin and streptomycin (100 IU/mL), and 10% fetal bovine serum (FBS). The ASFV isolate GZ201801 was propagated on PAMs and titrated using a hemadsorption (HAD) assay following the Reed Muench method, as previously described [32].

### Reagents

The primary antibody, mouse monoclonal antibody p30, was generated and stored in our laboratory. The secondary antibody, Alexa Fluor 568-conjugated goat anti-mouse IgG (H + L), was purchased from Cell Signaling Technology (Danvers, MA, USA). Horseradish peroxidase (HRP)-labeled goat anti-mouse IgG (A0216), β-actin mouse monoclonal antibody (AF0003), cell counting kit-8 (C0037), the DAB horseradish peroxidase color development kit (P0203), and the BCA protein assay kit (P0012s) were obtained from the Beyotime Institute of Biotechnology (Shanghai, China). Genistein was reported to possess potent anti-ASFV activity in vitro and was used as a positive control [12]. Brequinar, genistein, and uridine (purity ≥ 98%) were obtained from Chengdu Chroma-Biotechnology Co., Ltd. (Chen du, China). FerroOrange, Mito-FerroGreen, and Liperfluo were purchased from Dojido Laboratories (Kumamoto, Japan).

### Cell cytotoxicity assay

The cytotoxicity of brequinar on PAMs was evaluated using CCK-8 assay. Briefly, PAMs were seeded in 96-well plates and incubated for 4 h until they adhered to the plate wall completely. They were then exposed to increasing concentrations of brequinar (25–400 μM, six replicates) and cultured for 48 h. Next, 10 μL CCK-8 was added and the sample was incubated for 1 h at 37°C. Subsequently, optical density (OD) values were measured using a microplate reader (Thermo Fisher Scientific, MA, USA) at 450 nm. Untreated cells were considered 100% viable cells. The relative cell viability was calculated from the mean OD values of six wells per treatment. The 50% cytotoxic concentration (CC_50_) was analyzed using GraphPad Prism 8.0 (GraphPad Software, San Diego, CA).

### Antiviral activity assay

An antiviral activity assay was performed for brequinar to evaluate its capacity to inhibit ASFV replication. The PAMs were cultured in 24-well plates for 4 h and ASFV solution (MOI = 1) in essential medium was added before the supernatants were removed. After incubation for 2 h, the unabsorbed virus was removed, and fresh culture medium containing the compound at a 2-fold serial dilution was added. At the indicated time points, the viruses were collected and titrated using HAD, quantitative real-time PCR (qRT-PCR), and western blotting assays.

### Indirect immunofluorescence assay

Briefly, the cells were fixed with 4% paraformaldehyde for 10 min and permeabilized with 0.25% Triton X-100 for 10 min at 37℃. After blocking with 3% bovine serum albumin for 1 h at 37℃, the immobilized cells were incubated with ASFV p30 antibody (1:500) at 4°C overnight, washed with PBS, and incubated with Alexa Fluor 568 (1:1000) at 37°C for 1 h. Finally, 2-(4-aminophenyl)-6-indolecarbamidine was used to stain the nuclei at 37°C for 10 min and the samples were observed using a Leica DMI 4000 B fluorescence microscope (Leica, Wetzlar, Germany). The fluorescence ODs (blue and red, respectively) of each well were digitized using Image J software. The Normalized OD values (%) from the compound-treated samples were compared to those from the corresponding DMSO control groups (set as 100%). Protection percentage from compound-treated sample = [(100 − Normalized OD of compound-treated sample)/Normalized OD of compound-treated sample blue] × 100%. The EC_50_ value (the concentration required to protect 50% of cells from ASFV infection) was determined by plotting the protection percentage as a function of the compound concentration and calculated using a nonlinear regression function with GraphPad Prism software 8.0.

### Time-of-addition assay

The PAMs were seeded in 24-well plates for 4 h before the assay. For pre-treatment, the cells were treated with brequinar 2 h before ASFV infection (MOI = 1); For co-treatment, the cells were treated with brequinar and ASFV for 2 h; For post-treatment, the cells were infected with ASFV for 2 h and then treated with the brequinar. The cells were collected at 48 h post-infection and detected by western blotting assay.

### Direct interaction assay

PAMs were cultured in 24-well plates for 4 h. As previously published [33], ASFV (MOI = 1) and brequinar were mixed at 37°C for 1 h, and then separation of ASFV and brequinar by ultrafiltration centrifugation. ASFV particles collected in the ultrafiltration tube were washed twice with pre-cooled fresh culture medium, and then resuspended and added to the PAMs. After 48 h, the cells were collected and detected by western blotting assay.

### Uridine reversal assay

In the uridine reversal experiments, PAMs in plates were infected with ASFV (MOI = 1) for 2 h and then treated with 100 μM brequinar and uridine (12.5, 25, and 50 μM). After 24 h, the virus was collected and analyzed by qRT-PCR and western blotting assays.

### Confocal microscopy

PAMs in plates were infected with ASFV (MOI = 1) for 2 h and then treated with 100 μM brequinar. After 24 h, the cells were stained with FerroOrange, Mito-FerroGreen or Liperfluo, and then the fluorescence was visualized using TCS SP8 confocal microscope (Leica, Wetzlar, Germany).

### Quantitative real-time PCR

Total RNA was isolated from ASFV-infected PAMs with a total RNA rapid extraction kit (Fastagen, Shanghai, China) according to the manufacturer’s instructions, and reversely transcribed to first-strand cDNA using a reverse transcription kit (TaKaRa, Japan). PCR amplification was performed on 1 μL of template cDNA with primers. The primer sequences used in this study are listed in Table 1. qRT-PCR was completed using the CFX96 Real-time PCR system (Bio-Rad, USA) with 2 × RealStar Green Power Mixture containing SYBR Green I Dye (Genstar, Beijing, China). GAPDH was used as an endogenous control.

**Table 1:** List of primer sequences used in this study

Target	Sequence (5′–3′)	Orientation
ASFV-B646L	ATAGAGATACAGCTCTTCCG	Forward
ASFV-B646L	GTATGTAAGAGCTGCAGAC	Reverse
PAMs-GADPH	CCTTCCGTGTCCCTACTGCCAAC	Forward
PAMs-GADPH	GACGCCTGCTTCACCACCTTCT	Reverse

### Western blotting assay

Total cells were suspended in RIPA lysis buffer (Beyotime Biotechnology, Shanghai, China) on ice and standardized for protein content using a bicinchoninic acid kit (Beyotime, Biotechnology, Shanghai, China). The protein samples were separated using 10% sodium dodecyl sulphate-polyacrylamide gel electrophoresis and transferred to poly (vinylidene fluoride) membranes. The membranes were incubated with specific antibodies overnight at 4°C after blocking with 5% nonfat dry milk, followed by appropriate secondary antibodies. The membranes were imaged using a Tanon-5200 multi-infrared imaging system (Shanghai Tianneng Technology Co., Ltd.).

### Statistical analysis

The results are presented as the mean ± standard deviation of at least three independent experiments. The statistical significance between two groups was determined by Student’s *t*-test and that between more than two groups by one-way analysis of variance. *P* values < 0.05 were considered statistically significant.

## Results

### Evaluation of the cytotoxicity of brequinar

**Fig. 1:**
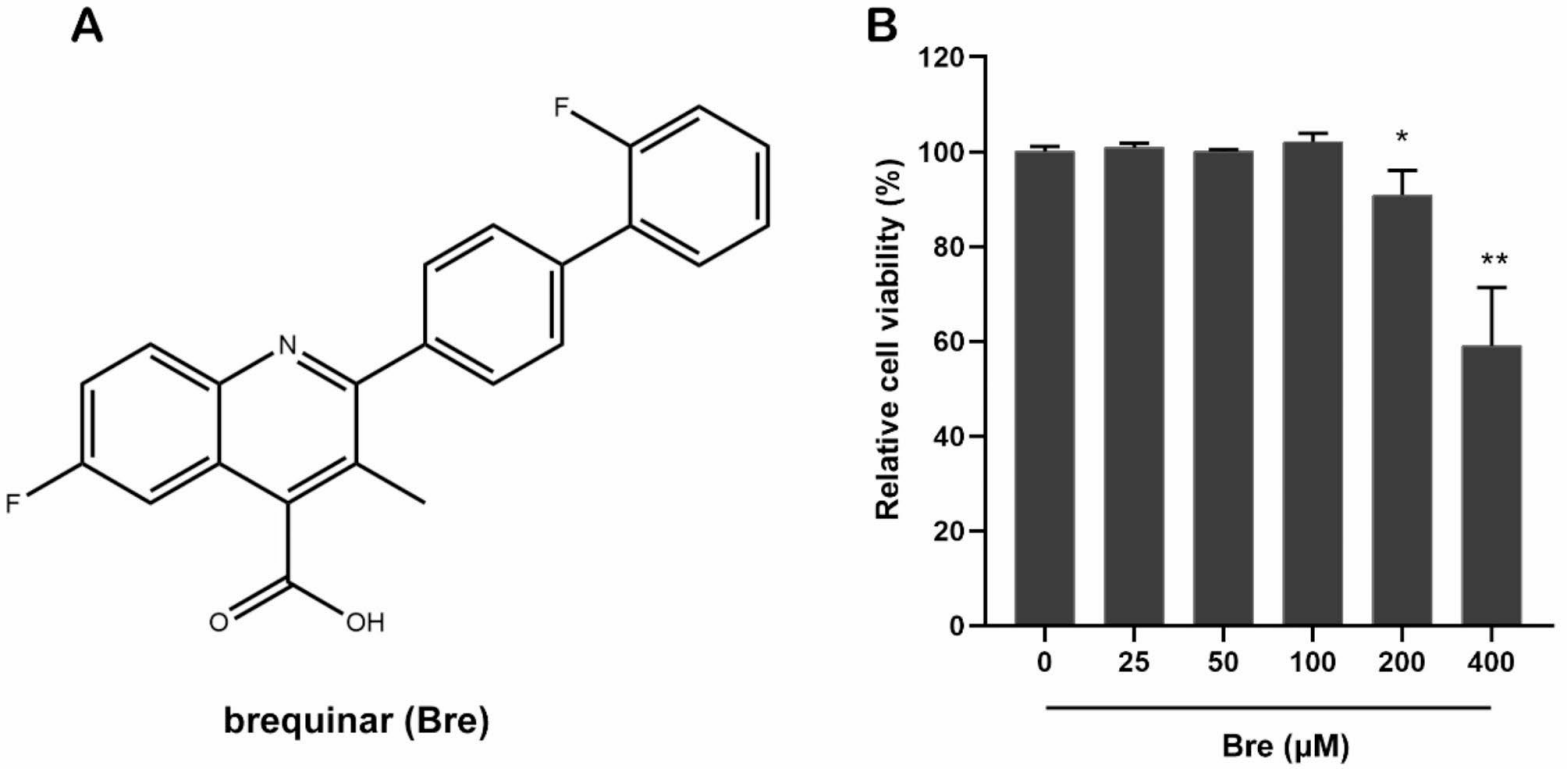
Cell viability of brequinar in PAMs. (**A**) The chemical structure of brequinar. (**B**) The cellular toxicity of brequinar in PAMs was evaluated by CCK-8 assay at 48 h post brequinar treatment. The relative viability of PAMs cultured in the absence of brequinar was set to 100%. Statistical significance is denoted by *P < 0.05 and **P < 0.01

The cytotoxicity of brequinar (Fig. 1A) towards PAMs was analyzed using CCK-8 assay. As shown in Fig. 1B, 400 μM brequinar significantly impaired the viability of PAMs, and the cell survival rate was approximately 59.7%. However, at concentrations from 25 to 100 μM, brequinar exhibited no cytotoxicity toward PAMs. The CC_50_ (reflecting 50% cell survival) of brequinar on PAMs was 451.8 μM.

### Antiviral effect of brequinar on ASFV infection in PAMs

**Fig. 2:**
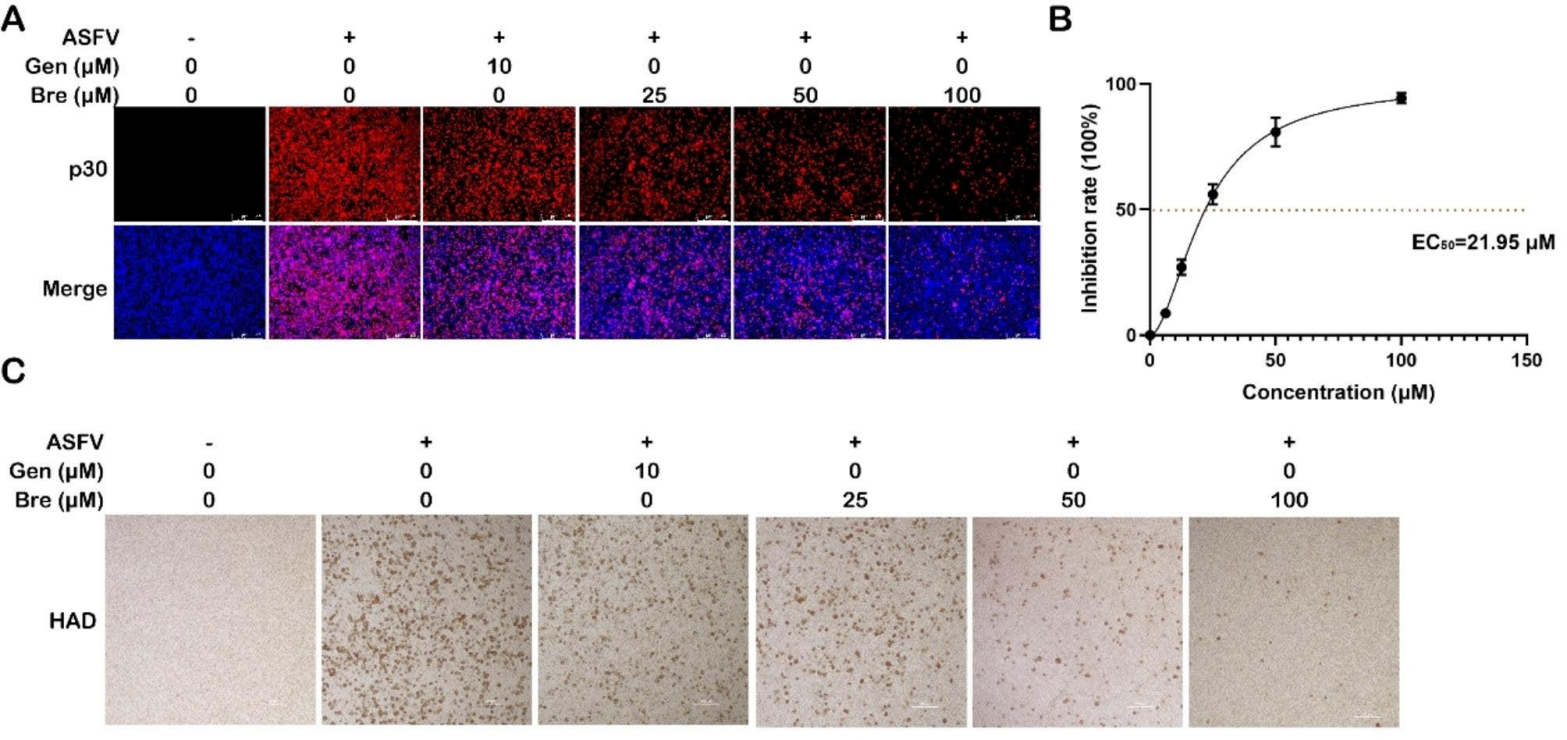
Brequinar inhibited ASFV replication in PAMs cells. The PAMs were infected with ASFV (MOI = 1) for 2 h, then fresh medium containing different concentrations of the compounds was added after the supernatants were removed. After incubating for 48 h, the samples were collected and detected by IFA assay (**A**) and HAD assay (**C**). (**B**) The EC_50_ value of brequinar was calculated based on IFA, as described in the methods

To investigate whether brequinar inhibits ASFV replication, we used different concentrations of brequinar to treat PAMs after ASFV infection. As shown in Fig. 2A, the results of an indirect immunofluorescence assay demonstrated that treatment with brequinar suppressed ASFV infection in a dose-dependent manner, and 100 μM of brequinar reduced the expression of ASFV p30 by over 90%. In addition, according to the results of IFA, we measured the fluorescence intensity of each sample using ImageJ software (NIH, Bethesda, MD, USA) and calculated the EC_50_ of brequinar to be 21.95 μM (Fig. 2B) using GraphPad Prism 8.0 software, and the selectivity index (SI, SI = CC_50_/EC_50_) was 20.58. Similarly, we found that brequinar suppressed the HAD induced by ASFV in a dose-dependent manner (Fig. 2C).

**Fig. 3:**
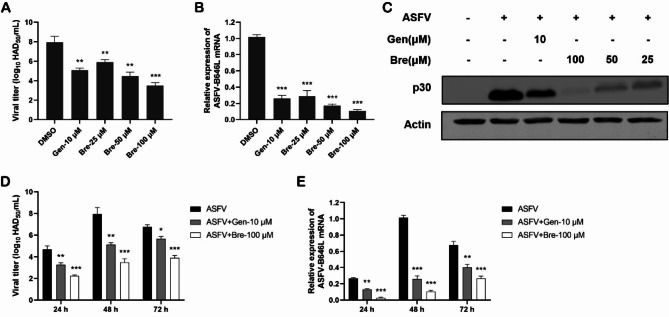
The antiviral effect of brequinar on ASFV replication in a dose- and time-dependent manner. PAMs were infected with ASFV (MOI = 1) for 2 h and then treated with compounds at various concentrations. At 48 h (**A-C**) or at indicated hours post-infection (**D, E**), samples were collected and detected by HAD_50_ assay, qRT-PCR assay, or western blotting assay. The virus titer was determined after treatment with brequinar for 48 h (**A**) or at the indicated hours post-infection (**D**). The expression level of ASFV-B646L mRNA was analyzed by qRT-PCR at 48 h (**B**) or at the indicated hours post-infection (**E**). The expression level of p30 protein was detected by western blotting assay after treatment with brequinar for 48 h (**C**). Statistical significance is denoted by *P < 0.05, **P < 0.01, and ***P < 0.001 compared to the DMSO-treated control

To confirm the anti-ASFV effect of brequinar, we examined the inhibitory effect of brequinar by HAD_50_, qPCR and western blotting (Fig. 3A, B and C, respectively). Remarkably, brequinar reduced the viral yield from 7.9 ± 0.5 log HAD_50_/mL to 3.4 ± 0.3 log HAD_50_/mL at 100 μM concentration (Fig. 3A). Simultaneously, brequinar downregulated both B646L mRNA levels and p30 protein levels by more than 90% compared to the DMSO-treated control (Fig. 3B and C, respectively). We further studied the ASFV inhibition kinetics of brequinar at 100 μM from 24 to 72 hpi by HAD_50_ and qPCR assays (Fig. 3D and E, respectively). As expected, the virus titers and B646L mRNA levels were reduced by treatment with brequinar at all time points, indicating that brequinar effectively inhibited multiple rounds of ASFV replication. Moreover, the reduction on ASFV replication of brequinar at 100 μM was stronger than that of the positive control genistein (Gen) at every time point. In summary, our results show that brequinar significantly inhibits ASFV infection.

### Brequinar inhibits ASFV infection in different treatment modes

**Fig. 4:**
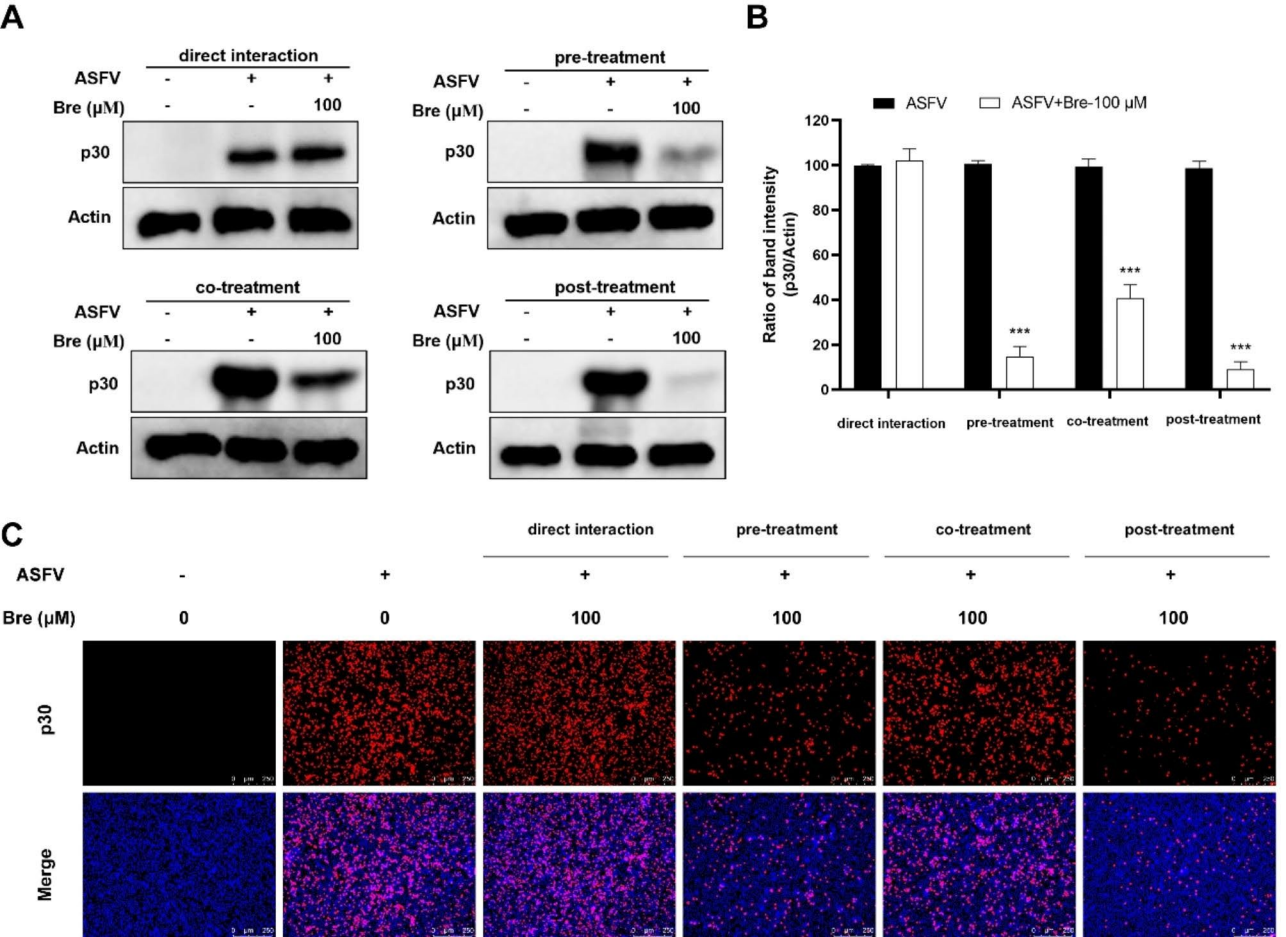
Brequinar inhibits ASFV infection in pre-, co-, and post-treatment. Brequinar was mixed with ASFV (MOI = 1) at 37℃ (direct interaction). After 1 h, the virus was separated from brequinar by ultrafiltration centrifugation and then resuspended to infect PAMs for 2 h. PAMs were treated with brequinar for 2 h prior to ASFV infection (pre-treatment). Brequinar was mixed with ASFV and then incubated for 2 h (co-treatment) on PAMs. PAMs were infected with ASFV for 2 h (post-treatment) and then treated with brequinar. After 48 h, the cells were harvested and detected by Western blotting (**A**) and IFA (**C**) assays. (**B**) The p30 and Actin band intensity was quantified using Image J software. Statistical significance is denoted by ***P < 0.001 compared to the DMSO-treated control

After demonstrating that brequinar exhibited potent inhibition against ASFV infection, we first explored whether brequinar interacted with ASFV directly. As shown in Fig. 4A, B and C, the direct interaction assay of brequinar with the virus did not suppress the expression of ASFV p30, suggesting that brequinar did not interact directly with ASFV. Subsequently, we investigated the potential mechanism of brequinar against ASFV via time-of-addition assay. We found that brequinar treatment reduced the expression of ASFV p30 in pre-, co-, and post-treatment, demonstrating that brequinar may exert anti-ASFV effects by modulating cellular antiviral components or by interfering with cellular components on which ASFV replication depends.

### Brequinar suppressed ASFV replication by activating ferroptosis

**Fig. 5:**
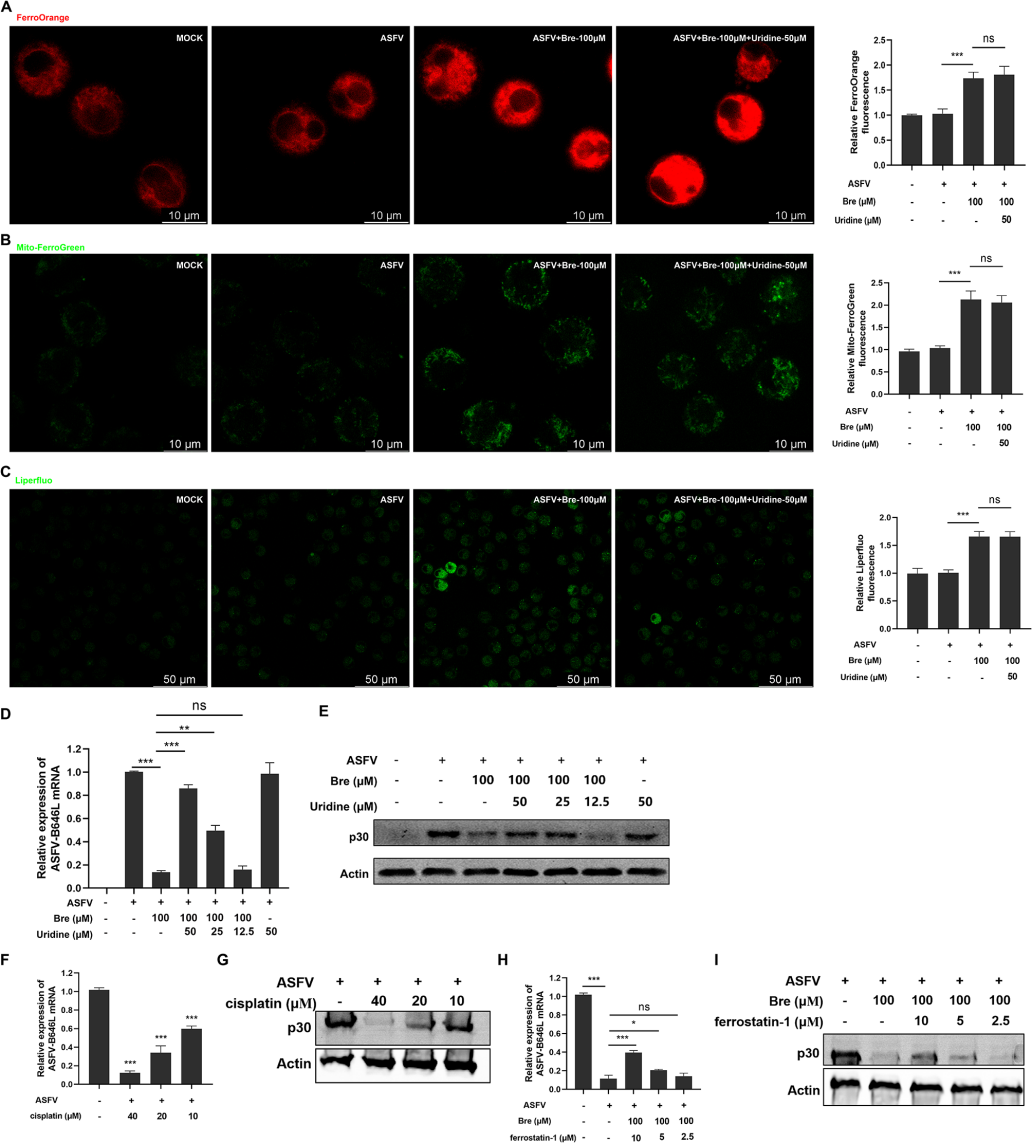
Brequinar suppressed ASFV replication by activating ferroptosis. (**A-C**) PAMs were infected with ASFV (MOI = 1) at 37℃ for 2 h, and then treated with 100 μM brequinar in the absence or presence of uridine. After 24 h, the cells were washed thrice, and incubated with FerroOrange (**A**), Mito-FerroGreen (**B**), or Liperfluo (**C**) for 30 min. The fluorescence was visualized with confocal microscopy. (**D, E**) PAMs were infected with ASFV (MOI = 1) for 2 h, then 100 μM brequinar was added in the absence or presence of uridine (12.5, 25, 50 μM) or 50 μM uridine was added alone. The plate was further incubated for 24 h, and then the ASFV-B646L mRNA level was analyzed by qRT-PCR assay (**D**) and the p30 protein level was detected by western blotting assay (**E**). (**F-I**) PAMs were infected with ASFV (MOI = 1) for 2 h, then treated with cisplatin (10, 20, 40 μM) (**F, G**) or 100 μM brequinar in the absence or presence of ferrostatin (2.5, 5, 10 μM) (**H, I**). The plate was further incubated for 24 h. The B646L mRNA and p30 protein level was detected by western blotting assay and qRT-PCR assay, respectively. Statistical significance is denoted by *P < 0.05, **P < 0.01, and ***P < 0.001

As previously reported, brequinar induced ferroptosis in cervical cancer [34]. However, little is known about whether brequinar exerts its antiviral effects through the induction of ferroptosis. Therefore, we investigated intracellular Fe^2+^, mitochondrial Fe^2+^, and lipid peroxides using FerroOrange, Mito-FerroGreen, and Liperfluo probes, respectively. As shown in Fig. 5A, B and C, ASFV infection did not alter the accumulation of intracellular Fe^2+^, mitochondrial Fe^2+^ or lipid peroxides compared to MOCK; however, they were increased by brequinar treatment. As is known to all, intracellular pyrimidine is important for RNA and DNA synthesis, brequinar reportedly exerts antiviral activity based on the inhibition of DHODH activity and the depletion of intracellular pyrimidine pools [35]. We checked whether brequinar inhibited ASFV replication by depleting intracellular pyrimidine pools. As shown in Fig. 5D and E, 100 μM brequinar significantly reduced the levels of ASFV-B646L and p30 proteins. Indeed, 25 and 50 μM uridine upregulated the levels of ASFV-B646L mRNA and p30 protein reduced by brequinar, indicating that uridine supplementation attenuated the anti-ASFV activity of brequinar. Treatment with 50 μM uridine alone had no effect on ASFV replication. Next, we further investigated whether supplementation with uridine could reverse the induction of Fe^2+^ or lipid peroxides by brequinar treatment. However, the results show that exogenous uridine supplementation did not alter the effect of brequinar on the induction of Fe^2+^ and lipid peroxides (Fig. 5A, B and C). Furthermore, we used the ferroptosis agonist cisplatin (Fig. S1) to investigate the effect of ferroptosis on ASFV replication. As shown in Fig. 5F and G, cisplatin inhibited the expression of ASFV B646l mRNA and p30 in a dose-dependent manner, respectively. Moreover, we found that the ferroptosis inhibitor ferrostatin-1 (Fig. S1) partially reversed the inhibitory effect of brequinar on ASFV, suggesting that the anti-ASFV effect of brequinar is dependent on ferroptosis (Fig. 5H ang I). Our results show that brequinar inhibits ASFV replication by activating ferroptosis, independent of inhibiting pyrimidine synthesis.

## Discussion

Almost a century has passed since the first outbreak of ASFV in Kenya, and it has caused huge economic losses to the global pork industry. More specifically, more than 1,000,000 pigs have been culled since August 3, 2018, owing to the lack of effective control measures [36]. Over the years, scientists have been working on developing vaccines against ASFV. However, there is currently no commercially available vaccine against ASFV [37]. In this context, we attempted to develop an antiviral drug therapy against ASFV. The DHODH inhibitor brequinar is a broad-spectrum antiviral inhibitor, we explored whether brequinar possesses anti-ASFV activity.

In this study, we demonstrated that brequinar strongly inhibits ASFV in a dose-dependent manner (Figs. 2 and 3) and sustain inhibition of ASFV from 24 to 72 hpi (Fig. 3). In general, the compounds exhibit antiviral activity in two ways: either the compound directly targets the virus itself, or the compound impairs host cell factors that are essential for the viral life cycle [38]. Therefore, we investigate whether brequinar interacts directly with ASFV thereby killing ASFV particles. However, the results showed that brequinar did not directly interact with ASFV; instead, brequinar inhibited ASFV replication in different treatment modes, including pre-, co-, and post-treatment, suggesting that brequinar inhibited ASFV replication by acting on cell factors (Fig. 4). DHODH, the fourth enzyme in the *de novo* pyrimidine biosynthesis pathway, is a popular target for antiviral and anticancer activities. Uridine is the precursor of the pyrimidine nucleotide. Exogenous uridine can be taken up by human ovarian cancer cell line 2008 cells and the uptake rate is essentially linear during the first 30 min [39]. Additionally, exogenous uridine has been used to supplement the consumption of endogenous uridine by the drugs in vitro and in vivo studies [40, 41]. As previously mentioned, brequinar exerted a broad-spectrum antiviral activity by inhibiting DHODH activity and depleting intracellular pyrimidine pools. Consistent with the reported results, we also found that exogenous supplementation with pyrimidines reversed the anti-ASFV activity of brequinar, demonstrating that brequinar suppressed ASFV replication by inhibiting DHODH activity and depleting intracellular pyrimidine pools (Fig. 5).

DHODH is thought to be an enzyme required for the *de novo* synthesis of pyrimidine nucleotides, but recently Mao et al. found that brequinar activates ferroptosis by inhibiting DHODH activity independently of GPX4 or FSP1 [30]. Ferroptosis is a recently discovered form of cell death characterized by massive iron accumulation and lipid peroxidation [42]. The virus-induced cell death has long been recognized as a double-edged sword that inhibits or exacerbates viral replication [43]. Cheng et al. found that SIV promotes viral replication by activating GPX4-mediated ferroptosis [44]. However, the effect of ASFV infection on ferroptosis and whether brequinar inhibits ASFV replication through ferroptosis are still unknown. Therefore, we investigated the impact of ASFV infection on ferroptosis and the results showed that ASFV infection did not induce ferroptosis; however, brequinar treatment induced ferroptosis and the accumulation of intracellular Fe^2+^, mitochondrial Fe^2+^ or lipid peroxides, which were not reversed by exogenous uridine supplementation. We further explored the effect of ferroptosis on ASFV replication and, as expected, treatment with the ferroptosis agonist cisplatin inhibited ASFV replication and found that the inhibitory effect of brequinar on ASFV was partially reversed by the ferroptosis inhibitor ferrostatin-1 (Fig. 5). Hence, the ferroptosis pathway may be a target for the development of anti-ASFV compounds.

It is important to note that there were some side effects with brequinar. Thrombocytopenia was the main side effect, but it was dose-limiting [45]. Given that all drugs have side effects, these clinical observations are acceptable. Importantly, the selectivity index > 20 of brequinar and the effective concentration of brequinar used in our study are much lower than the doses used in previous clinical trials [46]. Although inhibitors can negatively affect cellular function and may lead to deleterious long-term and broad consequences by targeting host cell signaling pathways, the safety and pharmacology of brequinar have already been tested in clinical trials [47, 48]. Moreover, Li and colleagues showed that brequinar inhibited FMDV replication and provided a 25% survival rate in FMDV-infected mice in vivo, suggesting that brequinar could be an effective anti-FMD antiviral agent [27]. Therefore, developing brequinar as an anti-ASFV inhibitor has advantages over developing new drugs or identifying new antiviral strategies.

## Conclusions

In summary, our data confirm that brequinar displays potent antiviral activity against ASFV in vitro and reveal the mechanism by which brequinar inhibits ASFV replication by activating ferroptosis, independent of inhibiting pyrimidine synthesis. Therefore, brequinar has potential as a novel drug or adjuvant therapeutic option to combat ASFV infection, and the ferroptosis pathway can be used as a novel target for anti-ASFV drug development.

### Electronic supplementary material

Below is the link to the electronic supplementary material.

Supplementary Material 1

Supplementary Material 2

## Data Availability

The datasets used and/or analyzed in this study are obtained and available from the corresponding authors upon a reasonable request.
