# Supplementary material for: Brequinar inhibits African swine fever virus replication in vitro by activating ferroptosis
Source: Virol J. 2023 Oct 24;20:242. doi: 10.1186/s12985-023-02204-x (PMC10599058; doi:10.1186/s12985-023-02204-x)
Supplement: Supplementary file 1 — Supplementary Material 1 [file 12985_2023_2204_MOESM1_ESM.docx]

Supplementary Material


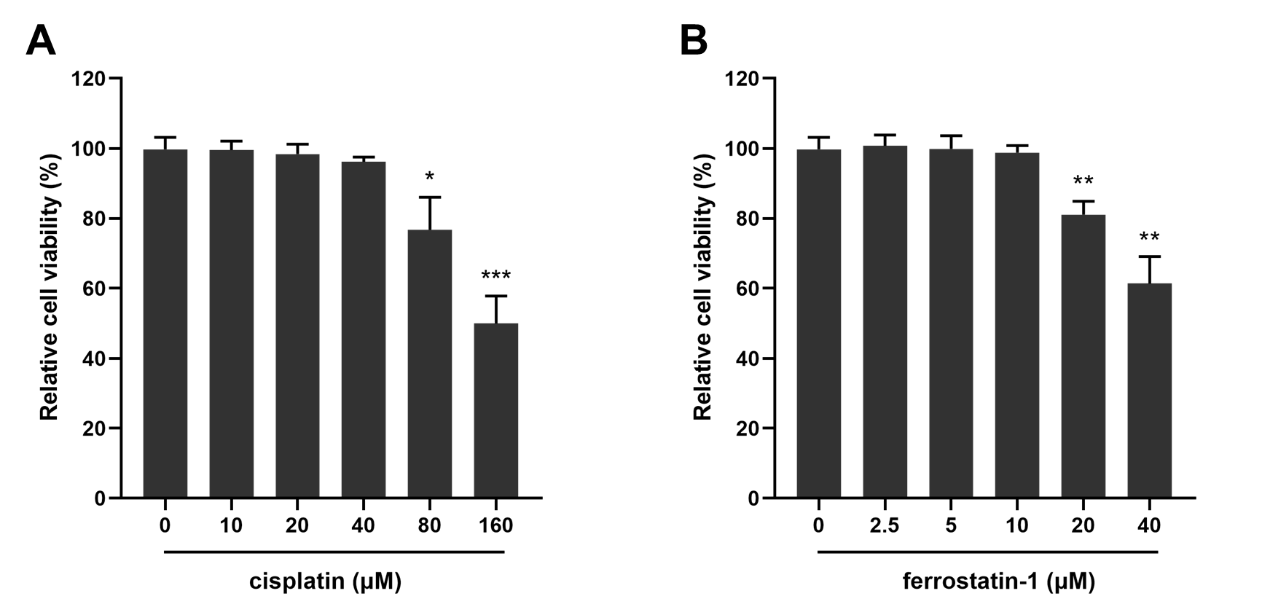


Fig. S1. Cell viability of cisplatin and ferrostatin in PAMs. The cellular toxicity of cisplatin (A) and ferrostatin (B) in PAMs was evaluated by CCK-8 assay at 24 h post cisplatin and ferrostatin treatment. **P* < 0.05, ***P* < 0.01, and ****P* < 0.001 represent statistical significance.
