# Supplementary material for: Brequinar inhibits African swine fever virus replication in vitro by activating ferroptosis
Source: Virol J. 2023 Oct 24;20:242. doi: 10.1186/s12985-023-02204-x (PMC10599058; doi:10.1186/s12985-023-02204-x)
Supplement: Supplementary file 2 — Supplementary Material 2 [file 12985_2023_2204_MOESM2_ESM.docx]

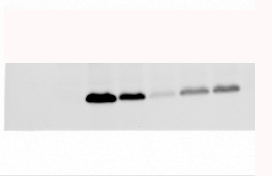


Fig. 3C (p30)


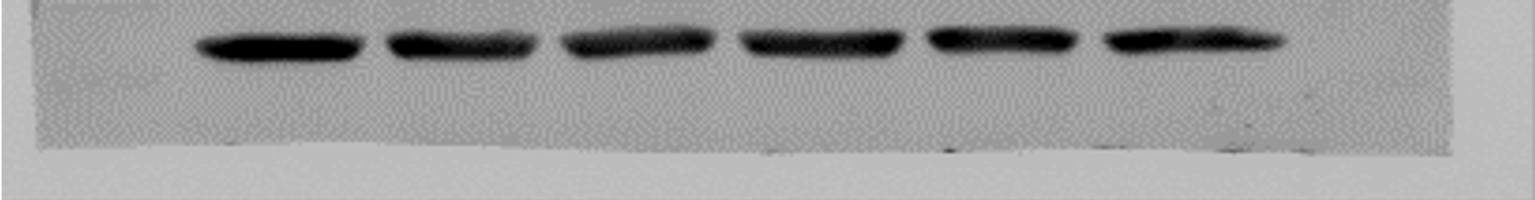


Fig. 3C (Actin)


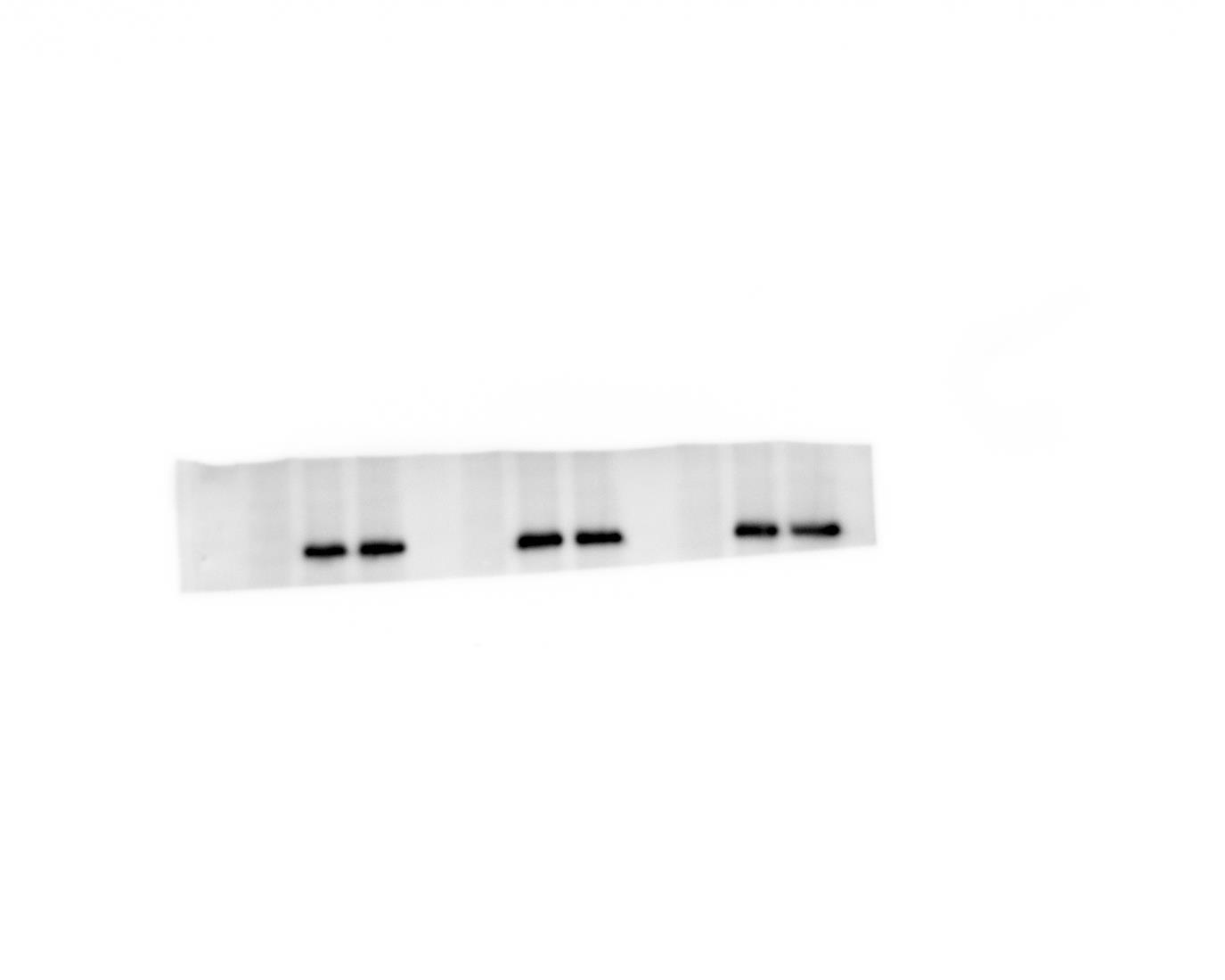


Fig. 4A (p30, direct interaction)


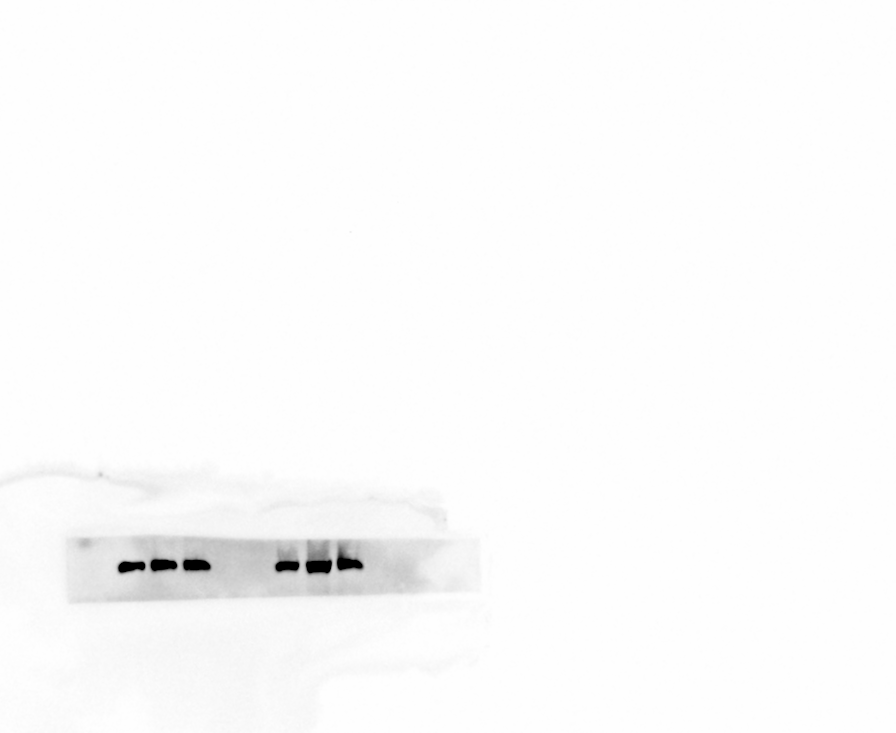


Fig. 4A (Actin, direct interaction)


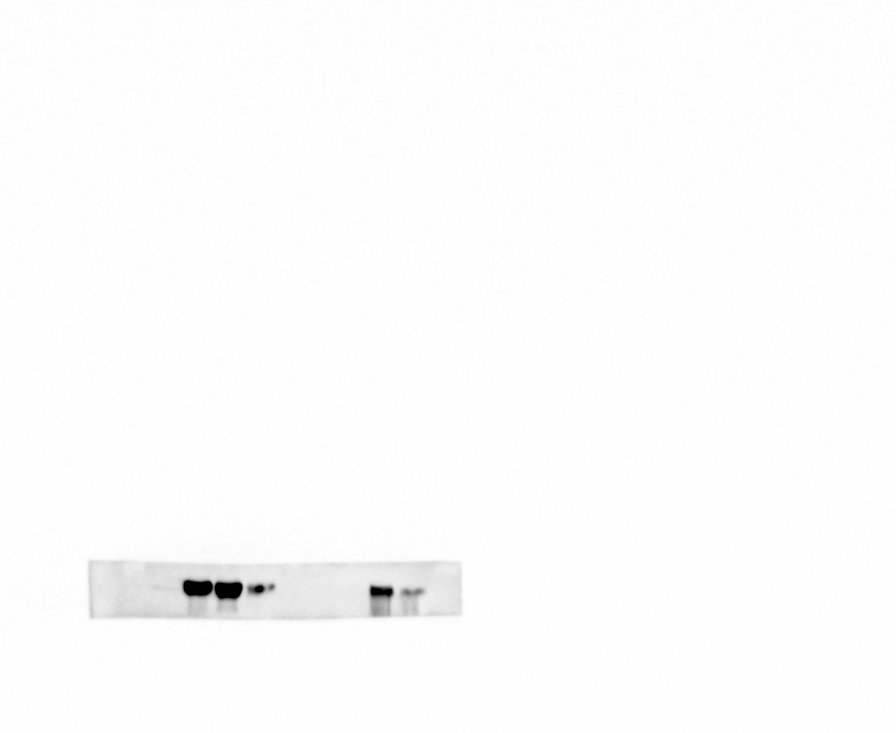


Fig. 4A (p30, pre-treatment)


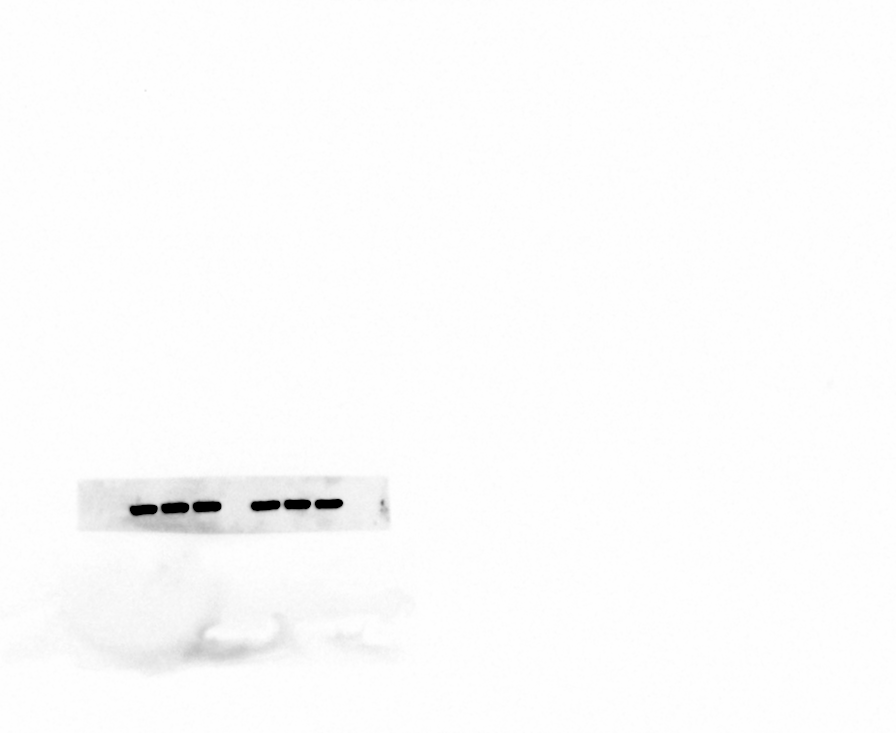


Fig. 4A (Actin, pre-treatment)


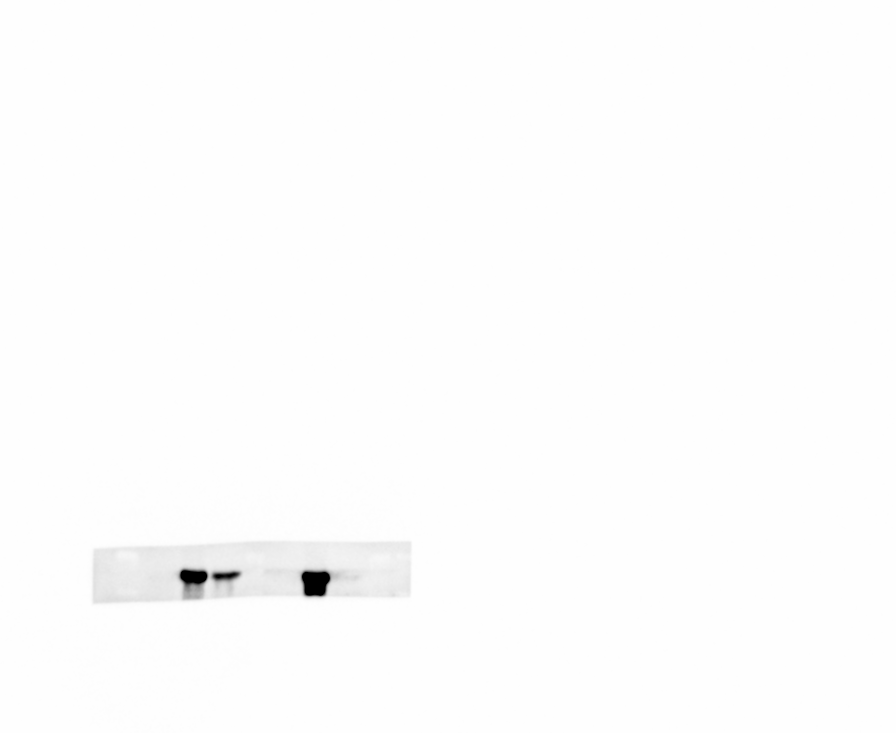


Fig. 4A (p30, co-treatment)


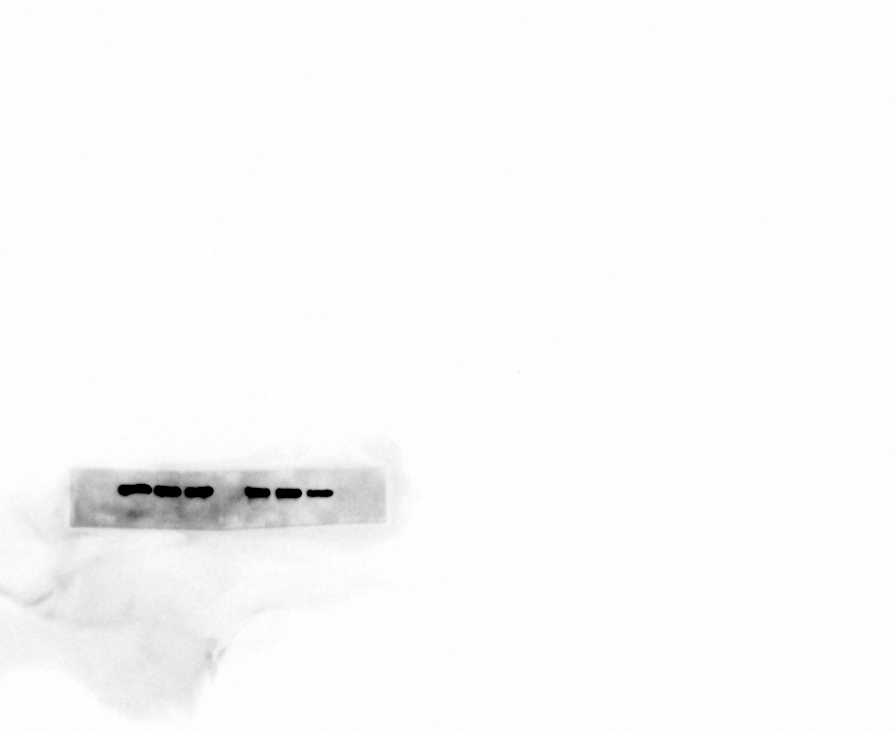


Fig. 4A (Actin, co-treatment)


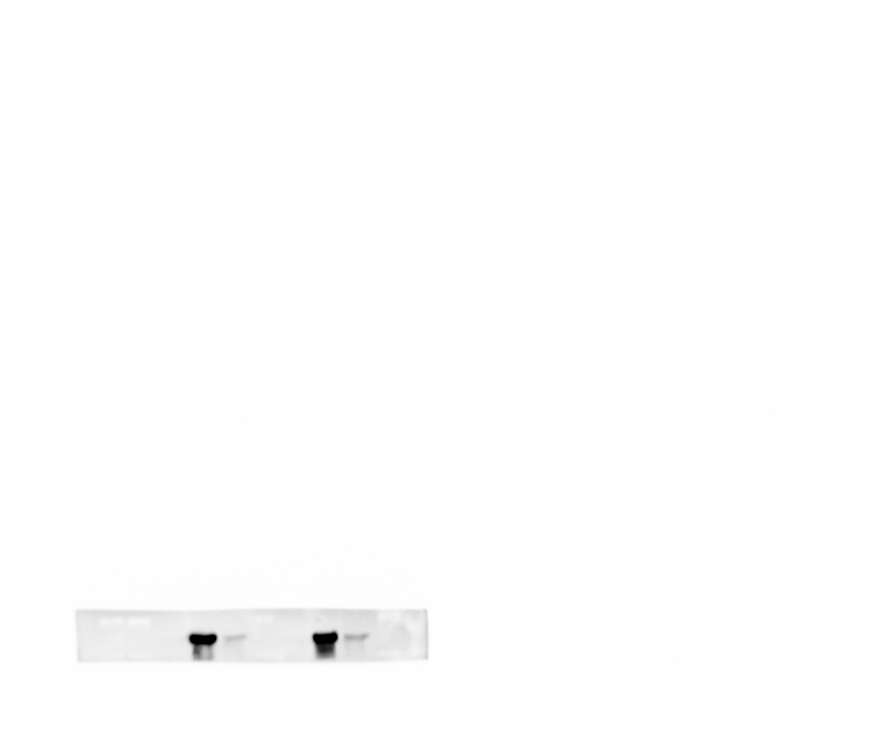


Fig. 4A (p30, post-treatment)


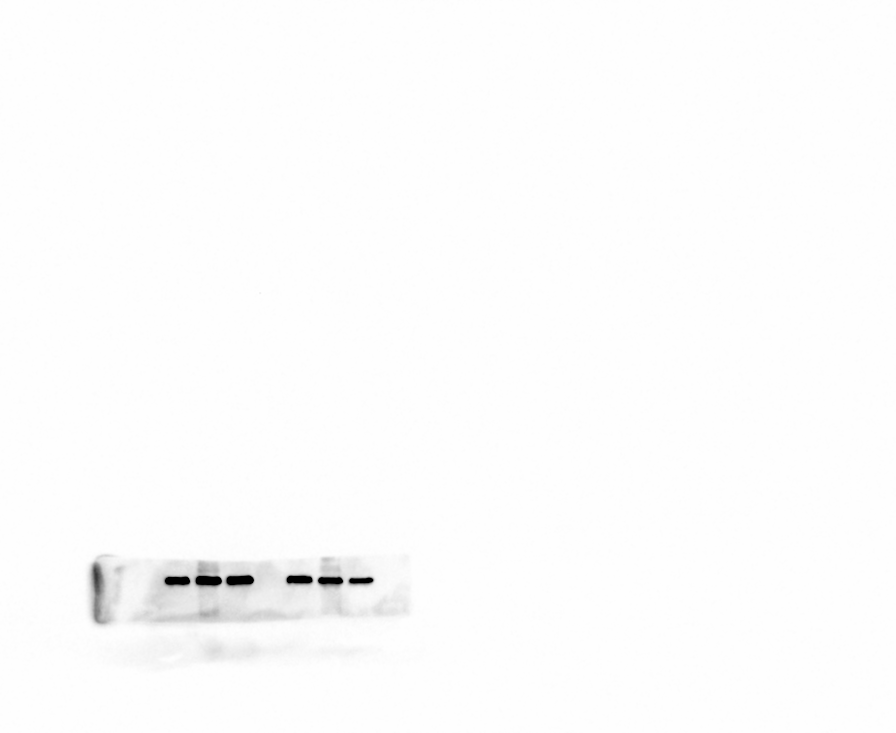


Fig. 4A (Actin, post-treatment)





Fig. 5E (p30)





Fig. 5E (Actin)


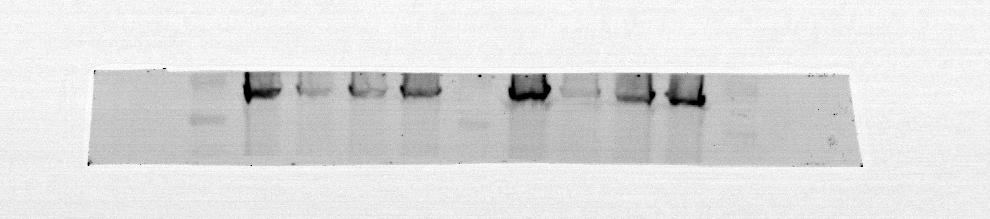


Fig. 5G (p30)


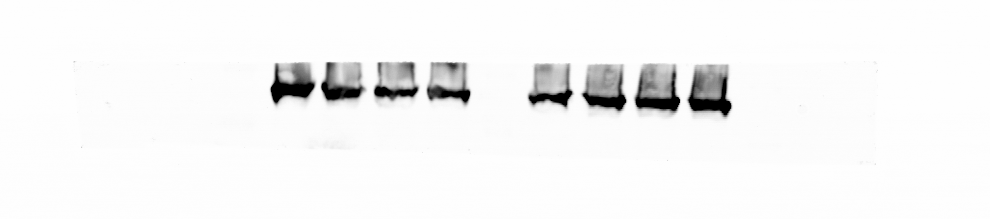


Fig. 5G (Actin)


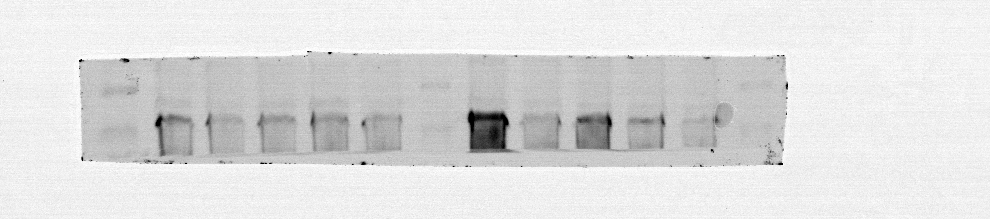


Fig. 5I (p30)


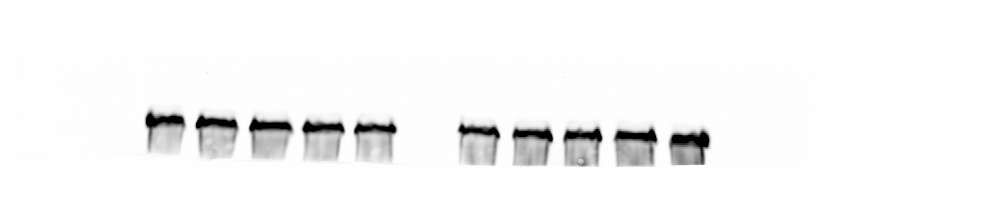


Fig. 5I (Actin)
